# Serum phosphoproteome alterations associated with cardiac troponin I levels in acute myocardial infarction

**DOI:** 10.55730/1300-0144.6194

**Published:** 2026-01-13

**Authors:** Merve ÖZTUĞ, Meltem AŞICIOĞLU, Konca ALTINKAYNAK, Evren KILINÇ

**Affiliations:** 1 TÜBİTAK National Metrology Institute, Kocaeli, Turkiye; 2 Department of Molecular Biology and Genetics, Faculty of Science and Letters, İstanbul Technical University, İstanbul, Turkiye; 3 Dr. Orhan Öcalgiray Molecular Biology-Biotechnology and Genetics Research Center, İstanbul Technical University, İstanbul, Turkiye; 4 Department of Medical Biochemistry, Erzurum Faculty of Medicine, University of Health Sciences, İstanbul, Turkiye; 5 Department of Biophysics, Hamidiye Faculty of Medicine, University of Health Sciences, İstanbul, Turkiye

**Keywords:** Myocardial infarction, proteomic analysis, phosphoproteins, biomarkers, mass spectrometry

## Abstract

**Background/aim:**

Acute myocardial infarction (AMI) triggers systemic biochemical responses, including dynamic changes in the phosphorylation status of circulating proteins. However, the phosphoproteomic profile of serum in the context of AMI remains insufficiently characterized. This study aimed to investigate serum phosphoproteomic alterations associated with AMI and to explore potential correlations with markers of cardiac injury.

**Materials and methods:**

A comparative phosphoproteomic analysis was performed on serum samples obtained from eight patients with AMI and pooled healthy control samples. High-abundance serum proteins were depleted, and phosphopeptides were enriched using TiO_2_ phosphopeptide enrichment kit. Samples were analyzed by liquid chromatography–tandem mass spectrometry using a Q Exactive HF-X Orbitrap mass spectrometer. Data were searched against the Homo sapiens database using Sequest HT with a 1% false discovery rate and were quantified by label-free quantification using Proteome Discoverer version 2.4.

**Results:**

A total of 46 phosphoproteins were confidently identified, revealing distinct phosphorylation profiles between AMI and control samples. Increased phosphorylation levels were observed for solute carrier family 12 member 5, apolipoprotein L1, the low-molecular-weight isoform of kininogen-1, and osteopontin in AMI serum. Conversely, phosphorylated inter-alpha-trypsin inhibitor heavy chain H2, antithrombin III, histidine-rich glycoprotein, peroxiredoxin-4, GTPase ERas, and the 26S proteasome non-ATPase regulatory subunit 1 were reduced or undetectable. A strong negative correlation was found between apolipoprotein L1 phosphorylation and cardiac troponin I concentrations (r = −0.91; p = 0.0016).

**Conclusion:**

These findings demonstrate that serum phosphoproteomics can provide valuable insights into the molecular events associated with AMI. The inverse relationship between apolipoprotein L1 phosphorylation and cardiac troponin I levels suggests that phosphoproteomic profiling may aid in understanding myocardial injury mechanisms.

## 1. Introduction

Acute myocardial infarction (AMI) is a leading cause of morbidity and mortality worldwide, resulting from the sudden disruption of coronary blood flow, most commonly due to atherosclerotic plaque rupture and thrombus formation [1,2]. Early and accurate diagnosis is critical to optimize clinical outcomes, and cardiac troponins—particularly cardiac troponin I (cTnI)—have become the gold standard for detecting myocardial injury due to their high specificity and sensitivity [3]. Serum cTnI levels are widely used to confirm AMI and to assess the severity of myocardial damage, as they correlate with infarct size, ventricular dysfunction, and clinical outcomes [4].

Despite their diagnostic value, troponins primarily reflect irreversible cellular injury and do not capture upstream regulatory or signaling events that precede necrosis [4]. This has driven interest in identifying circulating molecular signatures that reflect dynamic processes involved in myocardial injury. Among these, posttranslational modifications (PTMs), particularly phosphorylation, are gaining attention for their roles in modulating protein function, localization, and signaling pathways in cardiovascular disease [5,6].

To date, few studies have specifically profiled the serum phosphoproteome in AMI patients. Most proteomic research in cardiovascular disease has focused on tissues or cells rather than circulating proteins, due to the complexity and wide dynamic range of plasma components [7]. Nevertheless, foundational studies over the past decade have established methods to analyze phosphoproteins in human serum/plasma and provided baseline catalogs of phosphoproteins present in circulation. For example, early work by Carrascal et al. used immunodepletion of abundant proteins, strong cation-exchange fractionation, and titanium dioxide (TiO_2_) enrichment to identify 127 phosphorylation sites on 70 plasma proteins (false discovery rate [FDR] <1%) [8,9]. Notably, many of these phosphoproteins belonged to the complement system and the coagulation cascade [9], underscoring the relevance of phosphoregulation in inflammatory and clotting pathways. Subsequent large-scale efforts dramatically expanded the serum phosphoproteome. Jaros et al. analyzed serum from 80 individuals with immobilized metal affinity chromatography-based phosphoprotein enrichment, identifying over 5800 phosphopeptides mapping to 502 unique phosphoproteins [10]. This study demonstrated that a substantial proportion of circulating proteins, including low-abundance species, carry phosphorylation, with approximately 10% of sites occurring on tyrosine [10], representing a higher tyrosine proportion than that observed in typical cellular phosphoproteomes. These comprehensive datasets highlight that circulating phosphoproteins span diverse biological processes, including immune response, coagulation, and metabolism [9,10]. While no published study has yet focused exclusively on serum phosphoproteomic changes in AMI, it is likely that the acute inflammatory and thrombotic stress of myocardial infarction is reflected in altered phosphorylation of many plasma proteins. Indeed, global plasma proteomics in AMI has revealed shifts in proteins related to wounding, coagulation, and lipid metabolism [7], suggesting that phosphoproteomic analysis could uncover dynamic PTMs underlying these processes.

In light of these findings, serum phosphoproteomics emerges as a promising yet underexplored approach to characterize the systemic molecular responses to myocardial infarction. Given the involvement of phosphorylated plasma proteins in lipid transport, inflammation, and coagulation—all central to AMI pathophysiology—profiling the phosphoproteome of AMI patient serum may reveal novel regulatory events and candidate biomarkers. Therefore, in this study, we aimed to systematically profile phosphoproteins in the serum of patients with AMI using a label-free quantification (LFQ) liquid chromatography–tandem mass spectrometry (LC–MS/MS)-based workflow, and to explore potential associations between phosphorylation patterns and cardiac injury, as reflected by serum cTnI levels.

## 2. Materials and methods

### 2.1. Collection of serum samples

Blood samples were collected from patients diagnosed with AMI using yellow-capped biochemistry tubes at City Hospital, Erzurum, Türkiye. Serum was obtained by centrifugation at 4000 rpm at 4 °C for 5 min. Immunoassay measurements were performed immediately after sample collection, without freezing, using the ADVIA Centaur CP immunoassay system (Siemens Healthineers AG, Erlangen, Germany).

A total of eight patient serum samples were selected for phosphoproteomic analysis based on elevated cTnI concentrations, all exceeding the lower limit of quantification of our method. The measured cTnI levels were 5.81 μg/L, 7.84 μg/L, 8.34 μg/L, 10.20 μg/L, 12.36 μg/L, 16.52 μg/L, 16.62 μg/L, and 17.39 μg/L. In addition, serum samples obtained from 10 healthy individuals were pooled to generate a control sample. Following immunoassay testing, all serum samples (patient and control) were promptly frozen and stored at −80°C until LC–MS/MS analysis, thereby preserving sample integrity and minimizing protein degradation.

### 2.2. Serum protein extraction and immunodepletion

To facilitate the detection of differences in serum protein expression between AMI patients and healthy controls, highly abundant serum proteins were removed prior to phosphoproteomic analysis. On the day of analysis, serum samples from eight patients and two pooled control samples were processed. For each sample, 20 μL of serum was mixed with 2 μL of a 10X phosphatase inhibitor solution to preserve phosphorylation status. The inhibitor solution was prepared by dissolving one PhosSTOP phosphatase inhibitor tablet (F. Hoffmann-La Roche Ltd., Basel, Switzerland) in 1 mL of ultrapure water (18.2 M −.cm), resulting in a 10X stock solution.

Immunodepletion was performed using Pierce Top 12 Abundant Protein Depletion Spin Columns (cat. no: 85165; Thermo Fisher Scientific Inc., Waltham, MA, USA), following the manufacturer’s instructions. Each sample was processed through two spin columns, and the resulting flow-throughs were combined into a single tube to maximize depletion efficiency. The spin columns utilize immobilized antibodies targeting 12 highly abundant serum proteins, including albumin, immunoglobulins (IgG, IgA, and IgM), transferrin, fibrinogen, haptoglobin, alpha-1-antitrypsin, and apolipoproteins. This process efficiently removed more than 90% of the targeted proteins. Following immunodepletion, the flow-through was dried using a SpeedVac vacuum concentrator (Thermo Fisher Scientific Inc., Waltham, MA, USA), and the resulting protein pellet was reconstituted in 100 μL of 50 mM ammonium bicarbonate for further processing. Protein concentration was determined using a Qubit 4 Fluorometer (Thermo Fisher Scientific Inc., Waltham, MA, USA).

### 2.3. Preparation and enrichment of tryptic phosphopeptides

Approximately 300 μg of immunodepleted serum protein from each sample was subjected to enzymatic digestion using Rapigest SF surfactant (Waters Corporation, Milford, MA, USA), following the manufacturer’s protocol. Protein samples were adjusted to a final volume of 100 μL and then mixed with 100 μL of 0.2% RapiGest solution. To reduce disulfide bonds, 10 μL of 100 mM dithiothreitol was added (final concentration: 5 mM), and the samples were incubated at 60°C for 30 min. After cooling to room temperature, 36 μL of 100 mM iodoacetamide was added, resulting in a final concentration of 15 mM, and the samples were incubated in the dark at room temperature for 20 min. Digestion was performed by adding 5 μg of Trypsin/Lys-C Mix (Thermo Fisher Scientific Inc., Waltham, MA, USA), prepared in 0.1% acetic acid, to each sample (enzyme-to-protein ratio, 1:60). The digestion was carried out overnight at 37°C.

Following digestion, the peptide mixtures were dried using a SpeedVac vacuum concentrator (Thermo Fisher Scientific Inc., Waltham, MA, USA) and then reconstituted in 150 μL of binding buffer supplied with the Pierce High-Select TiO_2_ Phosphopeptide Enrichment Kit (cat. no: A32993; Thermo Fisher Scientific Inc., Waltham, MA, USA). Samples were loaded onto the spin columns and phosphopeptide enrichment was carried out according to the manufacturer’s protocol. Elution was performed using 2 × 50 μL of elution buffer. The eluates were combined, dried again using a SpeedVac vacuum concentrator (Thermo Fisher Scientific Inc., Waltham, MA, USA), and finally reconstituted in 25 μL of 95:5 (v/v) H_2_O:acetonitrile containing 0.1% formic acid. Phosphopeptides were stored at −80°C until LC–MS/MS analysis.

### 2.4. LC–MS/MS analysis

Phosphopeptide analyses were performed using a Q Exactive HF-X Orbitrap mass spectrometer coupled to an UltiMate 3000 RSLCnano UPLC system (Thermo Fisher Scientific Inc., Waltham, MA, USA). Peptides were first loaded onto an Acclaim PepMap C18 trap column (5 μm, 100 Å, 300 μm × 5 mm) and subsequently separated on an EASY-Spray RSLC C18 analytical column (2 μm, 100 Å, 75 μm × 25 cm) maintained at 40°C (Thermo Fisher Scientific Inc., Waltham, MA, USA). A 5 μL injection volume was used for each run, and peptides were separated at a flow rate of 350 nL/min using a reverse-phase gradient elution. Mobile phase A consisted of 0.1% formic acid in 98:2 H_2_O:ACN, and mobile phase B consisted of 0.1% formic acid in 98:2 ACN:H_2_O. The chromatographic gradient was applied over a total run time of 60 min and included the following steps: 3% to 8% B over 3 min, 8% to 24% B over 36 min, and 24% to 36% B over 12 min. This was followed by a rapid increase to 64% B in 0.5 min, held for 6.5 min, and reequilibrated with 3% B for 10 min.

The mass spectrometer operated in data-dependent acquisition mode using a “Full MS/dd-MS^2^” method. Full MS scans were acquired at a resolution of 60,000 (at m/z 200) over a scan range of m/z 350–1400, with an automatic gain control (AGC) target of 3E6 and a maximum injection time of 45 ms. MS/MS scans were triggered for the most intense ions, acquired at a resolution of 15,000 with an AGC target of 1E5, a maximum injection time of 22 ms, a 1.3 m/z isolation window, and an intensity threshold of 2E4. Fragmentation was performed with a normalized collision energy of 28%. Ionization was conducted in positive mode with a spray voltage of 2.0 kV, a funnel RF level of 50, and a capillary temperature of 270°C. Raw data files were deposited in the ProteomeXchange Consortium [11] via the PRIDE partner repository under the dataset identifier PXD062749 (DOI: 10.6019/PXD062749).

### 2.5. Data analysis

Raw data files were searched against the NCBI Homo sapiens protein database (taxonomy ID: 9606) using the Sequest HT search engine [12], with a strict FDR threshold of 0.01. Search parameters included a precursor mass tolerance of 10 ppm and a fragment mass tolerance of 0.02 Da. Carbamidomethylation of cysteine (+57.021 Da) was specified as a fixed modification, while oxidation of methionine (+15.995 Da), protein N-terminal acetylation (+42.011 Da), N-terminal methionine loss (−131.040 Da), N-terminal methionine loss with acetylation (−89.030 Da), and phosphorylation (+79.966 Da) on serine (S), threonine (T), and tyrosine (Y) residues were specified as variable modifications. Proteins were considered confidently identified if they matched at least two unique peptides, each with a minimum length of six amino acids. Protein quantification was performed using the LFQ module in Proteome Discoverer version 2.4 (Thermo Fisher Scientific Inc., Waltham, MA, USA). However, due to the low number of quantified background proteins (<500), automated statistical analysis was not performed within the software. Instead, we performed external statistical analysis of abundance values for each identified phosphoprotein across the control and AMI groups. Protein abundance distributions were first visually inspected, followed by nonparametric group comparisons using the Mann–Whitney U test, implemented in GraphPad Prism version 5.0 (GraphPad Software LLC, San Diego, CA, USA). Proteins with p values <0.05 were considered significantly differentially abundant between groups. Proteins showing a greater than two-fold change were considered significantly altered. To assess the biological relevance of the differentially abundant proteins, STRING functional enrichment analysis [13] was used to identify enriched biological processes, molecular functions, and signaling pathways.

## 3. Results

**Figure 1:**
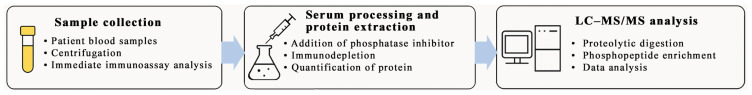
Overview of the experimental workflow used for serum phosphoproteomic profiling in AMI patients and healthy controls.

In this study, a label-free phosphoproteomic analysis was performed on serum samples collected from eight patients diagnosed with AMI and two pooled control samples generated from 10 healthy individuals. The workflow included immunodepletion of high-abundance proteins, enzymatic digestion, phosphopeptide enrichment, and LC–MS/MS-based identification and quantification. All samples were analyzed individually, and comparative phosphoproteomic profiles were generated for the AMI and control groups. Differentially phosphorylated proteins were identified using a 1% FDR threshold and subjected to functional annotation. The overall workflow of the study is illustrated in Figure 1.

**Figure 2:**
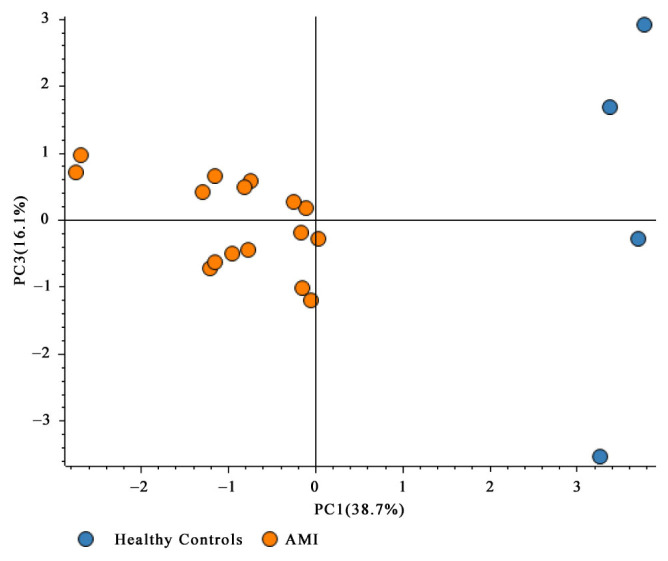
PCA of global serum phosphoproteomic profiles from AMI patients and healthy controls. The plot shows separation of samples along PC1 (38.7% of the variance) and PC3 (16.1% of the variance) based on normalized phosphoprotein abundance values. Each point represents an individual sample, colored according to clinical group (blue, healthy controls; orange, AMI patients). PCA was performed for exploratory visualization of global phosphorylation patterns.

A total of 132 protein groups and 878 peptide groups were identified across all serum samples analyzed. Among these, 46 phosphoproteins were confidently detected and quantified. Principal component analysis (PCA) was performed to visualize global variation in phosphoproteomic profiles between AMI and healthy control serum samples. As shown in Figure 2, the samples clearly clustered according to their respective groups, indicating a distinct separation between AMI and control samples based on their phosphorylation signatures. This separation suggests that the phosphoproteomic alterations observed are robust and group-specific, supporting the biological relevance of the differentially phosphorylated proteins identified.

A total of 10 phosphoproteins exhibiting a greater than two-fold change in abundance between the control and AMI groups were identified. These proteins, along with their corresponding phosphorylation sites and localization probabilities, are summarized in Table 1. Among these, solute carrier family 12 member 5 (Q9H2X9-1), apolipoprotein L1 (O14791), the low-molecular-weight (LMW) isoform of kininogen-1 (P01042-2), and osteopontin (P10451-1) showed altered phosphorylation status, with elevated phosphorylation levels in AMI serum. In contrast, inter-alpha-trypsin inhibitor heavy chain H2 (P19823), antithrombin III (P01008), histidine-rich glycoprotein (P04196), peroxiredoxin-4 (Q13162), GTPase ERas (Q7Z444), and the 26S proteasome non-ATPase regulatory subunit 1 (Q99460) were either significantly reduced or undetectable. Notably, some proteins demonstrated exclusive presence or absence in either group (e.g., infinite or zero ratios), reinforcing their potential utility as AMI-specific phosphoproteomic indicators. Statistical analysis was performed using the Mann–Whitney U test.

**Figure 3:**
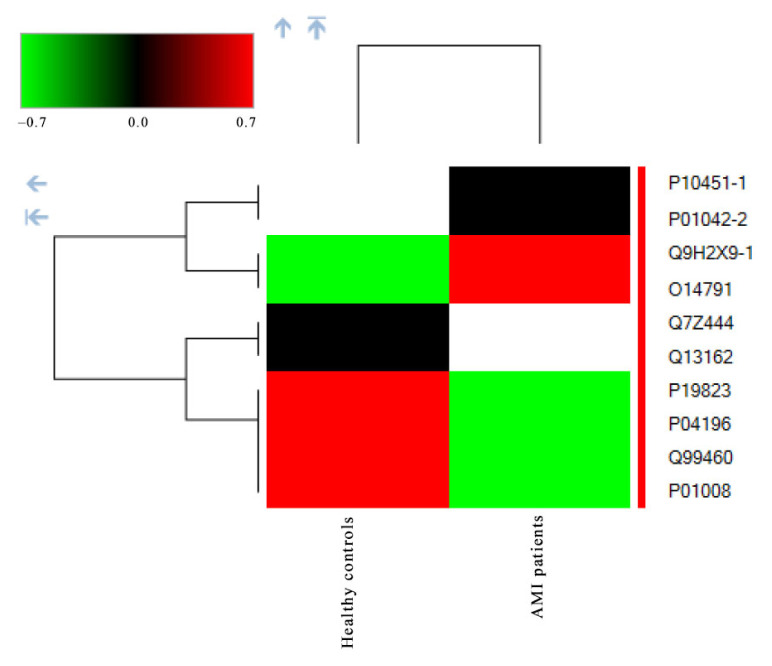
Heatmap showing relative phosphoprotein abundance in serum samples from patients with acute myocardial infarction (AMI) and healthy controls. Colors represent log_2_-transformed, normalized protein abundance values, with green indicating lower abundance and red indicating higher abundance. Black cells indicate proteins that were not detected under the corresponding condition. For each group, values represent the mean abundance across three technical replicates per extraction condition. Hierarchical clustering was applied to both proteins and sample groups.

To further explore abundance patterns and intersample variability, a heatmap visualization was generated for the quantified phosphoproteins. As shown in Figure 3, the heatmap revealed clear and consistent differences in phosphorylation levels between AMI and control samples, with green indicating low phosphorylation levels and red indicating high phosphorylation levels. Some phosphoproteins displayed uniform upregulation or downregulation across all AMI patients, including those highlighted in Table 1, supporting their potential biological relevance in disease-associated signaling pathways. Notably, proteins such as osteopontin and the LMW isoform of kininogen-1 were exclusively detected in AMI samples, whereas others, including peroxiredoxin-4 and the 26S proteasome subunit, were absent in the disease group, reinforcing their group-specific behavior. These distinct phosphorylation patterns may reflect key pathophysiological processes triggered during AMI.

Protein abundance values are represented as log_2_-transformed intensities, with green indicating low and red indicating high levels. Gray denotes proteins not detected in a given sample. Data are shown for eight AMI samples and two pooled healthy controls.

To explore the relationship between the phosphorylation state of serum proteins and the degree of myocardial injury, we performed a correlation analysis between phosphoprotein levels and serum cTnI concentrations. The cTnI levels were measured immediately after blood collection using the ADVIA Centaur CP immunoassay system (Siemens Healthineers AG, Erlangen, Germany), without prior freezing, to ensure accuracy and minimize degradation. Concentrations ranged from 5.81 μg/L to 17.39 μg/L across the eight AMI patient samples included in the phosphoproteomic analysis.

Phosphoprotein abundance values were obtained using LFQ. For each AMI sample, a relative abundance ratio was calculated by dividing its abundance value by the average abundance of the healthy control pool (n = 10). These normalized ratios were then used for Pearson correlation analysis against cTnI levels. This statistical method reflects the strength and direction of linear relationships between two variables, where r values closer to +1 or −1 indicate stronger correlations.

**Figure 4:**
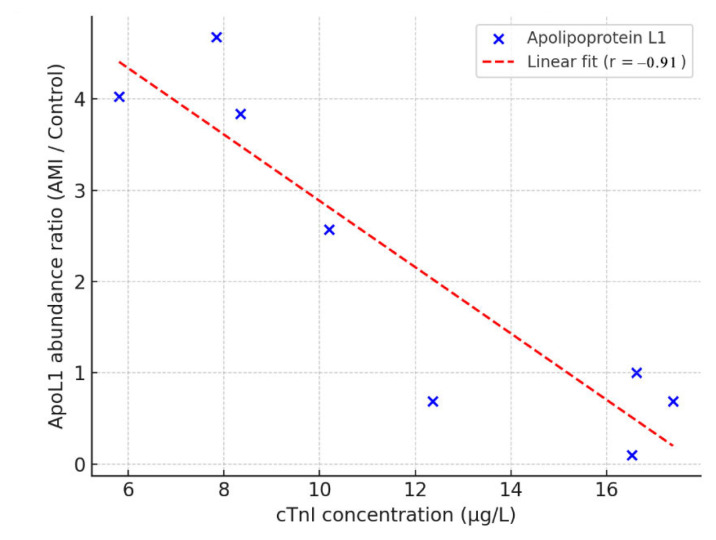
Scatter plot illustrating the inverse association between apolipoprotein L1 phosphorylation levels (normalized abundance) and serum cTnI concentrations in AMI patients. Pearson correlation analysis was used to assess the strength and direction of the linear relationship between variables (r = −0.91; p = 0.002). The regression line is shown for visualization purposes only and does not represent a predictive model.

The results of the correlation analysis are summarized in Table 2. Among the proteins analyzed, apolipoprotein L1 (ApoL1) exhibited a strong and statistically significant negative correlation with cTnI levels (r = −0.91; p = 0.002), as shown in Figure 4, where a clear inverse trend is observed across individual AMI samples. Although ApoL1 did not show a statistically significant difference between AMI and control groups (p > 0.05; Table 1), it was included in both Table 1 and the correlation analysis due to its notable interindividual variation. The mean abundance ratio (AMI/control) was 2.20, with a standard deviation of 1.69, and individual patient ratios were: 4.03, 4.68, 3.84, 2.57, 0.69, 0.10, 1.00, and 0.69. These data indicate that at least four patients exhibited more than a 3.5-fold increase, suggesting substantial upregulation in a subset of the AMI cohort. This variability likely reduced the statistical power in group-wise comparisons. Nevertheless, the significant inverse correlation with cTnI suggests that ApoL1 phosphorylation may reflect the extent of myocardial injury.

**Figure 5:**
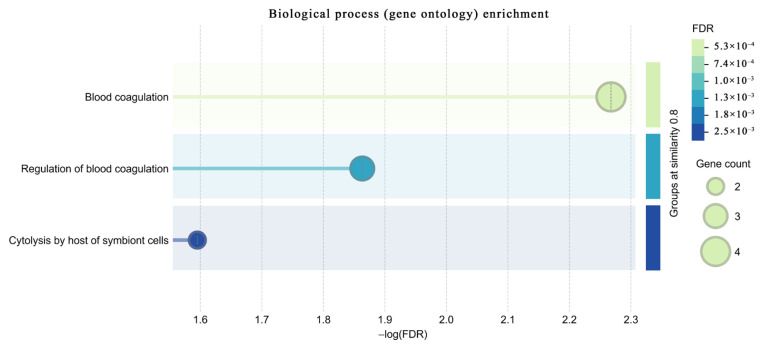
Functional enrichment analysis of differentially phosphorylated proteins identified in AMI serum. The x-axis represents the −log_10_ FDR-adjusted p value, indicating enrichment significance. Bubble size corresponds to the number of proteins associated with each functional term, whereas bubble color reflects the FDR value, with darker shades indicating higher statistical significance. Enrichment analysis was performed using STRING functional annotation.

Functional enrichment analysis of differentially phosphorylated proteins was performed using STRING (version 12) for Homo sapiens (taxonomy ID: 9606). The results revealed significant enrichment in blood coagulation–related processes, including “blood coagulation” (GO:0007596; p = 0.0054), “regulation of blood coagulation” (GO:0030193; p = 0.0137), and “cytolysis by host of symbiont cells” (GO:0051838; p = 0.0254). These functional insights are visually summarized in Figure 5, which highlights blood coagulation and immune regulatory processes as major biological themes among the differentially phosphorylated serum proteins in AMI patients. Additionally, molecular function categories such as “endopeptidase inhibitor activity” (GO:0004866; p = 2.57 × 10^−6^), “heparin binding” (GO:0008201; p = 3.51 × 10^−5^), and “glycosaminoglycan binding” were significantly enriched, indicating an overrepresentation of coagulation-regulatory and immune-modulatory proteins in the phosphoproteome of AMI patients. Importantly, Reactome pathway analysis identified “posttranslational protein phosphorylation” (R-HSA-8957275; p = 3.17 × 10^−10^) and “platelet degranulation” (R-HSA-114608; p = 0.022) among significantly enriched terms, reinforcing the relevance of these modifications in AMI pathology.

Reactome pathway enrichment of the differentially phosphorylated serum proteins revealed several pathways with strong relevance to acute AMI pathophysiology. The most significant enrichments were observed in posttranslational protein phosphorylation (p = 3.17 × 10^−10^), regulation of insulin-like growth factor transport (p = 4.28 × 10^−10^), and platelet degranulation (p = 0.022). These pathways are tightly linked to endothelial dysfunction, inflammatory signaling, and platelet activation, further supporting the role of altered phosphorylation dynamics in AMI.

## 4. Discussion

This study provides new insights into the serum phosphoproteomic alterations associated with AMI. By integrating high-confidence phosphopeptide enrichment with LFQ, we identified 46 distinct phosphoproteins and demonstrated marked differences between AMI patients and healthy controls. The altered phosphorylation profiles suggest the activation of biological pathways related to lipid transport, coagulation, and inflammation—all of which are fundamental to the pathophysiology of AMI.

Taken together, these findings support a conceptual framework in which circulating phosphoproteins reflect upstream regulatory and systemic responses to myocardial injury, complementing conventional biomarkers that capture irreversible cardiomyocyte damage.

Cardiac troponin I (cTnI) is widely recognized as a reliable and sensitive biomarker for myocardial injury and remains the clinical gold standard for the diagnosis of AMI. Its concentration in blood increases in response to cardiomyocyte necrosis, making it a direct indicator of myocardial cell damage [2]. However, as cTnI reflects irreversible structural damage, it is primarily considered a late-phase marker [14]. Integrating cTnI with upstream molecular indicators—such as PTMs—may improve patient stratification and offer deeper insights into the molecular mechanisms underlying AMI pathophysiology.

In this context, our finding of a strong inverse correlation between ApoL1 phosphorylation levels and serum cTnI concentrations (r = −0.91; p = 0.0016) is particularly noteworthy. ApoL1 is a high-density lipoprotein (HDL)-associated protein with recognized roles in lipid metabolism and innate immunity, and is inducible by proinflammatory cytokines such as interferon-γ and TNF-α, both of which are elevated in AMI [14]. ApoL1 has been found to be phosphorylated at multiple serine residues (S327, S330, S352, and S355) [16], yet the functional implications of these modifications remain unclear. Given its localization on HDL particles, phosphorylation may affect its distribution, ligand interactions, or vascular signaling roles during acute ischemia. While more studies are needed to define its mechanistic contributions, our data suggest that ApoL1 phosphorylation may serve as an indicator of systemic responses to myocardial injury. Its inverse relationship with cTnI supports a potential complementary role in capturing early or regulatory phases of myocardial damage, distinct from the structural cell death reflected by cTnI itself. In this context, ApoL1 phosphorylation may reflect early or regulatory systemic responses to myocardial stress rather than direct cardiomyocyte necrosis, highlighting its potential value as a complementary molecular indicator alongside cTnI.

Beyond ApoL1, several other phosphoproteins demonstrated AMI-associated regulation. Solute carrier family 12 member 5 was significantly elevated in AMI serum and has not previously been described in cardiac phosphoproteomic studies. While its functional role in cardiovascular disease remains unclear, this transporter is involved in ion balance and neuronal excitability [17], and its presence in serum may reflect systemic stress responses or vascular remodeling. LMW kininogen, an alternatively processed isoform of kininogen-1, is generated by proteolytic cleavage and lacks the bradykinin sequence present in its high-molecular-weight counterpart. LMW kininogen primarily functions as a protease inhibitor and participates in the regulation of fibrinolysis and inflammatory responses. Although its role in AMI has not been extensively characterized, its exclusive presence and phosphorylation in AMI serum, as observed in this study, may reflect compensatory mechanisms aimed at limiting excessive proteolysis or modulating vascular responses during myocardial injury [18–20].

Antithrombin III (AT, SERPINC1) is the principal endogenous anticoagulant, neutralizing thrombin and factor Xa, particularly in the presence of heparan sulfate or therapeutic heparin. Recent phosphoproteomic analyses have identified phosphorylation sites such as T63 and S68 in its amino-terminal region [16], suggesting potential modifications during maturation. While the exact functional consequences of AT phosphorylation remain unclear, antithrombin plays a central role in balancing coagulation and inflammation by inactivating thrombin and dampening its proinflammatory signaling through protease-activated receptors [21]. Inflammatory conditions, such as sepsis, are known to reduce AT levels and activity, exacerbating coagulopathy [21]. In the context of AMI, localized thrombin generation and systemic inflammation are prominent, and phosphorylation of AT may influence its anticoagulant function or interaction with heparin, thereby impacting vascular integrity and thrombo-inflammatory responses.

Histidine-rich glycoprotein (HRG) is a multifunctional 75-kDa plasma protein that binds heparin, fibrin, plasminogen, phospholipids, and metal ions [22]. Through these interactions, HRG regulates coagulation, fibrinolysis, immune responses, and angiogenesis. It modulates macrophage polarization, promoting proinflammatory (M1) phenotypes while inhibiting antiinflammatory (M2) responses [23]. Decreased HRG levels have been associated with impaired immune–coagulative balance in inflammatory conditions such as sepsis, and HRG supplementation has been shown to improve endothelial function and survival [22,23]. Although specific phosphorylation sites on HRG have not been well characterized [16], its structural flexibility suggests that phosphorylation could influence its ligand binding and vascular regulatory functions during AMI.

In addition to these proteins, our phosphoproteomic analysis identified several others of interest. Inter-alpha-trypsin inhibitor heavy chain H2, an extracellular matrix-associated protein, showed decreased phosphorylation in AMI serum, possibly reflecting extracellular matrix remodeling or protease regulation during myocardial injury [24]. Peroxiredoxin-4, a secreted antioxidant enzyme involved in redox homeostasis, was absent in AMI serum samples, suggesting oxidative stress-induced depletion or altered secretion dynamics [25]. The non-ATPase regulatory subunit 1 of the 26S proteasome, a key component of the ubiquitin–proteasome system essential for protein degradation, exhibited a loss of detectable phosphorylation, which may indicate systemic disturbances in proteostasis or vesicle-mediated clearance under ischemic stress [26]. GTPase ERas, a Ras family member involved in cell survival and proliferation signaling, was similarly undetectable in AMI serum, possibly reflecting suppression of survival pathways during myocardial ischemia [27]. Osteopontin, a multifunctional phosphoprotein involved in inflammation, wound healing, and fibrosis, was exclusively detected and phosphorylated in AMI serum, supporting its proposed role as a mediator of postinfarction inflammatory and fibrotic remodeling [28]. Collectively, these phosphorylation changes point to coordinated modulation of coagulation, inflammatory signaling, oxidative stress, and proteostasis during AMI, emphasizing that systemic molecular adaptations extend beyond cardiomyocyte injury alone.

Pathway enrichment analysis using STRING revealed significant clustering of the differentially phosphorylated proteins in biological processes such as blood coagulation, glycosaminoglycan binding, and platelet degranulation, reinforcing the relevance of the identified phosphoproteins to the thrombo-inflammatory milieu of AMI. Cellular localization terms such as blood microparticle and vesicle lumen were also enriched, consistent with the notion that extracellular vesicles and secretory granules contribute to the release of phosphorylated proteins into circulation during AMI. The enrichment of vesicle- and microparticle-associated terms further supports the translational relevance of circulating phosphoproteins as accessible indicators of systemic thrombo-inflammatory activity in AMI.

## 5. Limitations

This study has several limitations that should be acknowledged. First, the relatively limited sample size restricts statistical power and generalizability. However, this work was intentionally designed as an exploratory, hypothesis-generating phosphoproteomic investigation rather than a population-level validation study. Serum phosphoproteomic analysis is inherently resource-intensive and costly, particularly when performed using immunodepletion of high-abundance proteins and phosphopeptide enrichment strategies combined with high-resolution LC–MS/MS, as applied in the present study. These methodological requirements substantially increase per sample analytical costs and limit the feasibility of large-cohort analyses at the discovery stage, especially under restricted research budgets. Despite this limitation, the study revealed biologically coherent phosphoproteomic alterations and a strong inverse correlation between apolipoprotein L1 phosphorylation and serum cTnI concentrations, supporting the relevance of the identified candidate proteins. Importantly, the phosphoproteins highlighted in this study represent testable targets that can be evaluated in larger, independent cohorts using more cost-effective and high-throughput analytical approaches, such as immunoassay-based methods (e.g., ELISA), once appropriate assays become available. Future studies employing scalable validation platforms and expanded patient populations will be essential to determine the translational and clinical relevance of these phosphorylation-based markers.

## 6. Conclusion

In conclusion, this study provides a phosphoproteomic overview of human serum in the context of AMI, highlighting the dynamic regulation of circulating proteins through phosphorylation during cardiac injury. Using LFQ mass spectrometry, we identified distinct serum phosphoproteomic profiles that differentiate AMI patients from healthy controls. Among these, the inverse correlation observed between ApoL1 phosphorylation and serum cTnI concentrations is particularly noteworthy. While cTnI remains the gold standard for diagnosing myocardial infarction, it reflects irreversible cellular damage; in contrast, phosphoproteins such as ApoL1 may capture upstream or regulatory processes associated with myocardial stress and systemic inflammation. These findings suggest that serum phosphoproteomic profiling could complement existing biomarkers and provide additional mechanistic insights into AMI pathophysiology. However, further validation in larger, well-characterized cohorts, including longitudinal sampling and functional studies, will be essential to clarify the clinical relevance, translational potential, and mechanistic roles of these phosphorylation-based markers in myocardial injury and repair.

## Figures and Tables

**Table 1:** Phosphoproteins exhibiting ≥2-fold increases or decreases in AMI serum compared with healthy controls.

Accession	Description	Ratio	p-value	Modifications
**P19823**	Inter-alpha-trypsin inhibitor heavy chain H2	0.36	0.004	Phospho [S60(99)]
**Q13162**	Peroxiredoxin-4	−∝	NA	Phospho [S140(99.2)]
**P04196**	Histidine-rich glycoprotein	0.13	0.003	Phospho [S356(100)]
**P01008**	Antithrombin III	0.21	0.004	Phospho [S68(100)]
**O14791**	Apolipoprotein L1	2.20	NS	Phospho [S311(100); S314(100)]
**Q9H2X9-1**	Solute carrier family 12 member 5	3.66	0.003	Phospho [T883(100)]
**Q99460**	26S proteasome non-ATPase regulatory subunit 1	−∝	NA	Phospho [T273(95.2); S277(93.6)]
**P01042-2**	LMW isoform of kininogen-1	+∝	NA	Phospho [T327(98.9); S332(100);S406(100)]
**Q7Z444**	GTPase ERas	−∝	NA	Phospho [T16(96.3)]
**P10451-1**	Osteopontin	+∝	NA	Phospho [S224(100); S234(100)]

Values of +∝ or −∝ indicate phosphorylation sites detected exclusively in one group (+∝ in AMI or −∝ in controls), meaning that the corresponding phosphopeptides were consistently detected in one condition but not detected in the other. These values therefore reflect condition-specific phosphorylation patterns rather than quantitative fold changes. NS: not significant; NA: not applicable.

**Table 2:** Pearson correlation coefficients between phosphoprotein abundance and cTnI levels.

*Protein*	*Pearson r*	*p-value*
*Apolipoprotein L1*	−0.91	0.002
*Histidine-rich glycoprotein*	−0.57	0.139
*Antithrombin III*	−0.41	0.316
*Solute carrier family 12 member* 5	−0.19	0.645
*Inter-alpha-trypsin inhibitor heavy chain* H2	−0.13	0.752
*26S proteasome non-ATPase* regulatory subunit 1		
*GTPase ERas*		
*Osteopontin*		
*Peroxiredoxin-4*		
*LMW* isoform *of* k*ininogen-1*		
